# Effects of Dapagliflozin vs. Vildagliptin on the Lipid Profile of Patients With Uncontrolled Type 2 Diabetes

**DOI:** 10.7759/cureus.89091

**Published:** 2025-07-30

**Authors:** Shreshth Khanna, Mayank Malik, Razi Ahmad

**Affiliations:** 1 Pharmacology and Therapeutics, Hamdard Institute of Medical Sciences and Research, New Delhi, IND; 2 Pharmacology, GS Medical College and Hospital, Pilkhuwa, IND

**Keywords:** dapagliflozin, diabetes, dpp-4 inhibitors, sglt2 inhibitor, vildagliptin, vildagliptin and metformin treatment

## Abstract

Background

Type 2 diabetes (T2D) represents one of the most common metabolic disorders globally. Insulin resistance is a fundamental issue associated with the disorder, wherein cells in the adipose tissue, liver, and muscle resist the action of insulin, leading to dysregulation of glucose metabolism. T2D is a known, significant, and independent risk factor for the development and progression of dyslipidemia, a condition characterized by abnormal lipid levels in the blood. Inadequately controlled T2D can lead to dyslipidemia through various mechanisms. Dyslipidemia, i.e., alteration in the levels of plasma lipids typically characterized by increased levels of triglycerides (TGs), total cholesterol (TC), low-density lipoprotein cholesterol (LDL-C), and low levels of high-density lipoprotein cholesterol (HDL-C), is a significant and modifiable risk factor in the development of atherosclerotic cardiovascular disease (ASCVD).

Materials and methods

In this prospective, observational, parallel-group, open-label study, 383 patients of inadequately controlled T2D receiving metformin (500-2000 mg) were randomized to receive vildagliptin (group A) or dapagliflozin (group B) for a 24-week duration. We compared the effects of vildagliptin and dapagliflozin on hemoglobin A1c (HbA1c), fasting plasma glucose (FPG), and lipid profile at baseline and after 24 weeks of therapy.

Results

A total of 248 patients with T2D completed the follow-up for 24 weeks (mean age: 53.8±8.4 years, and the mean body mass index {BMI} was 25.0±3.4 kg/m^2^). From the baseline to 24 weeks, the HDL-C increased by 1.1 mg/dL in group A and by 3.17 (95% CI: 3.0-7.1) mg/dL in group B (p=0.03).

A significant decrease in the mean LDL-C levels by 11.8 (95% CI: -2.10, -16.6) mg/dL was observed in group A, whereas an increase in the levels of mean LDL-C by 6.4 (95% CI: 6.1, 6.69) mg/dL in group B (p=0.03) was observed. There was no significant difference in the mean change from baseline in the HbA1c levels (-0.71 vs. -1.08, p=0.65) and in the levels of TC (p=0.87), TG (p=0.37), apolipoprotein A (p=0.986), apolipoprotein B (p=0.563), and lipoprotein(a) (p=0.767) between the vildagliptin and the dapagliflozin group after 24 weeks of therapy.

Conclusions

In conclusion, both vildagliptin (a DPP-4i) and dapagliflozin (an SGLT2i), when added to metformin, showed comparable efficacy in controlling plasma glucose in patients with inadequately controlled type 2 diabetes. However, they had differing effects on lipid profiles and body weight. Vildagliptin led to greater reductions in LDL-C, suggesting its preference in patients with elevated LDL-C levels. In contrast, dapagliflozin may be more suitable for obese patients with low HDL-C. Thus, adding either drug to metformin not only improves glycemic control (FPG and HbA1c) but also favorably influences lipid profiles, potentially reducing ASCVD risk in this population.

## Introduction

T2D is a chronic, progressive metabolic disorder involving multiple organ systems. It is typically characterized by elevated blood glucose levels, impacting various macrovascular and microvascular systems, including the heart, blood vessels, eyes, kidneys, and nerves. Globally, approximately 540 million people are estimated to be affected by type 2 diabetes (T2D), with around 100 million residing in the Indian subcontinent [1]. The overall prevalence of T2D is expected to affect 800 million people by 2045 [2].

The existing literature highlights T2D as an independent risk factor for various cardiovascular disorders, including increased risk of dyslipidemia, atherosclerosis, and coronary and peripheral arterial diseases. Diabetic dyslipidemia (DD), with an estimated prevalence of over 90%, is one of the common complications following insulin resistance encountered in patients with uncontrolled T2D [3].

Patients with DD, besides sustained hyperglycemia, i.e., fasting plasma glucose (FPG) ≥126 mg/dL, random glucose levels ≥200 mg/dL, and HbA1c levels ≥6.5%, have an elevation in the levels of triglycerides (TGs), low-density lipoprotein cholesterol (LDL-C), and a reduction in the levels of high-density lipoprotein cholesterol (HDL-C), which are significant modifiable risk factors in cardiovascular disease. Although uncommon, euglycemia with oral anti-diabetic drugs (OADs) alone might alleviate the risk of dyslipidemia in patients with T2D [3]. Insulin plays a pivotal role in regulating the plasma glucose levels. Alterations in the plasma insulin levels and insulin resistance directly contribute to altered lipid metabolism via de novo lipogenesis, inhibition of lipolysis, and ectopic fat deposition [4].

The existing guidelines endorse lifestyle modifications accompanied by metformin alone as the introductory step in the management of T2D. The disease progression is invariably accompanied by decreased insulin production and/insulin resistance, altered lipid metabolism, dyslipidemia, which can lead to atherogenesis and elevated risk of atherosclerotic cardiovascular disease (ASCVD) [5]. Many cases of T2D with suboptimal blood glucose control necessitate the use of second-line OADs or switching to insulin-based regimens [6].

The dipeptidyl peptidase-4 inhibitors (DPP-4i) and the sodium-glucose co-transporter-2 inhibitors (SGLT2i), due to their insulin-independent actions, established efficacy, tolerability, and low hypoglycemia risk, along with their additional lipid-lowering and pleiotropic effects on cardiovascular and renal systems, are now commonly contemplated as add-on drugs to metformin [7]. Dapagliflozin, an SGLT2i, is effective both as a monotherapy and in combination with other OADs. It helps in lowering the blood glucose levels by inhibiting the SGLT2 receptors in the proximal tubules of the kidney, leading to increased glucose excretion without affecting insulin secretion [8].

Vildagliptin, a DPP-4i, acts by decreasing the plasma DPP-4 activity by up to 80% and elevating active glucagon-like peptide-1 (GLP-1) levels, thus enhancing the cell functionality [9]. Several studies have shown promising results for vildagliptin to effectively reduce plasma lipid levels in T2D patients [10].

Since these agents are widely considered second-line agents in addition to metformin, primarily in diabetic patients with a low risk of ASCVD or chronic kidney disease (CKD) progression, we planned a study to assess the efficacy and safety of dapagliflozin and vildagliptin when combined with metformin in patients with inadequately controlled T2D with dyslipidemia.

## Materials and methods

The study population included patients with inadequately controlled T2D who were initiated on treatment with either dapagliflozin or vildagliptin in addition to metformin between March 2021 and May 2022. The present study was a prospective, observational, parallel-group, open-label study conducted over 24 weeks at the Department of Pharmacology, in association with the Department of General Medicine, Hamdard Institute of Medical Sciences and Research, Jamia Hamdard, New Delhi, India.

Before enrolling the first patient, we obtained approval from the Institutional Ethics Committee, Hamdard Institute of Medical Sciences and Research, Jamia Hamdard, New Delhi (#HIMSR/IEC/018/2021, dated March 19, 2021). This study was conducted in accordance with the Declaration of Helsinki and the Good Clinical Practice guidelines. The patient information sheet, describing the study's objective, attributable risks, and possible benefits, was provided in the local language to patients screened for eligibility.

The eligible patients included individuals who had been diagnosed with T2D with inadequately controlled blood glucose despite combined diet/exercise and metformin monotherapy for more than 12 weeks before screening. The main inclusion criteria were as follows: (1) age ≥18 years, (2) diagnosed with T2D at least three months before screening, (3) HbA1c ≥7.5% and ≤9% on treatment, and (4) willing to provide written informed consent to participate.

Patients with type 1 diabetes; a history of pre-existing heart failure, myocardial infarction, deep vein thrombosis, thromboembolic stroke, chronic kidney disease (CKD), pancreatitis, or any other chronic disorder within the last three months; a history of changes in prior medications that could influence lipid profile - including HMG-CoA reductase inhibitors (statins), fibrates, niacin or nicotinic acid, omega acid esters, thyroid medications, or steroids - within 12 weeks before recruitment or during treatment with vildagliptin or dapagliflozin; an estimated glomerular filtration rate (eGFR) of ≤60 mL/min/m²; fasting serum triglycerides ≥600 mg/dL; or elevated serum bilirubin levels at screening, as well as pregnant and lactating women, were excluded from the study.

Eligible patients were enrolled and assigned via computer-generated randomization in a 1:1 ratio to receive either dapagliflozin or vildagliptin in addition to metformin for 24 weeks. Randomization was performed with the use of a computer-generated random-sequence number in a block-of-4 randomization manner.

Patients randomized to group A received the tablet vildagliptin 50 mg orally once a day before meals, the doses were increased gradually to 50 mg twice daily till the conclusion of the study, whereas the patients randomized to group B received a tablet of dapagliflozin 10 mg orally once daily before meals, which was gradually augmented to 10 mg twice daily, until the study conclusion. No crossover of the study medications was allowed. Patients from both treatment groups were treated with tablet metformin 500-2000 mg per day in single or divided doses. The treating physician was responsible for the dose alterations.

At the baseline visit, the sociodemographic parameters were recorded in the case record form. All the patients underwent general physical and systemic examinations. Blood specimens were drawn to ascertain various hematological parameters, liver function tests, and lipid profiles of patients. Throughout the study, the privacy of the participants and data confidentiality were thoroughly ensured.

Fasting blood samples, including the levels of FPG, HbA1c, TC, TG, HDL-C, LDL-C, liver function tests (LFT) including aspartate aminotransferase (AST) and alanine transaminase (ALT), were measured and evaluated at baseline visit and 24 weeks after DPP-4i or SGLT2i combination therapy.

Statistical analysis

Assessments in this study were strictly limited to the per-protocol (PP) population. To establish the appropriate sample size, calculations were performed using the expected mean HbA1c difference, derived from prior studies. All data were expressed using appropriate descriptive statistics, including mean±standard deviation, frequencies, medians with interquartile ranges, or 95% confidence intervals. For inferential analysis, categorical variables were compared using the chi-square test. Intergroup and intragroup analyses were conducted using the unpaired t-test and repeated measures ANOVA, respectively.

The Wilcoxon signed-rank test was applied to variables that did not meet the assumptions of normal distribution. Specifically, the impact of DPP-4i and SGLT2i treatment on HbA1c and serum lipids from baseline to 24 weeks was analyzed via paired t-tests, while changes in TG and HDL-C levels were assessed using the Wilcoxon signed-rank test.

The effect of vildagliptin and dapagliflozin on lipid profiles from baseline to 24 weeks was assessed using Analysis of Covariance (ANCOVA). This statistical model included treatment as the primary factor while controlling for potential confounding variables, such as age, sex, diabetes duration, BMI, and the percentage change in HbA1c. For analyses, a p-value of less than 0.05 was considered statistically significant. All the data analysis was performed using the latest version of SPSS Statistics version 31 (Chicago, IL: IBM Corp.) [11].

## Results

**Figure 1:**
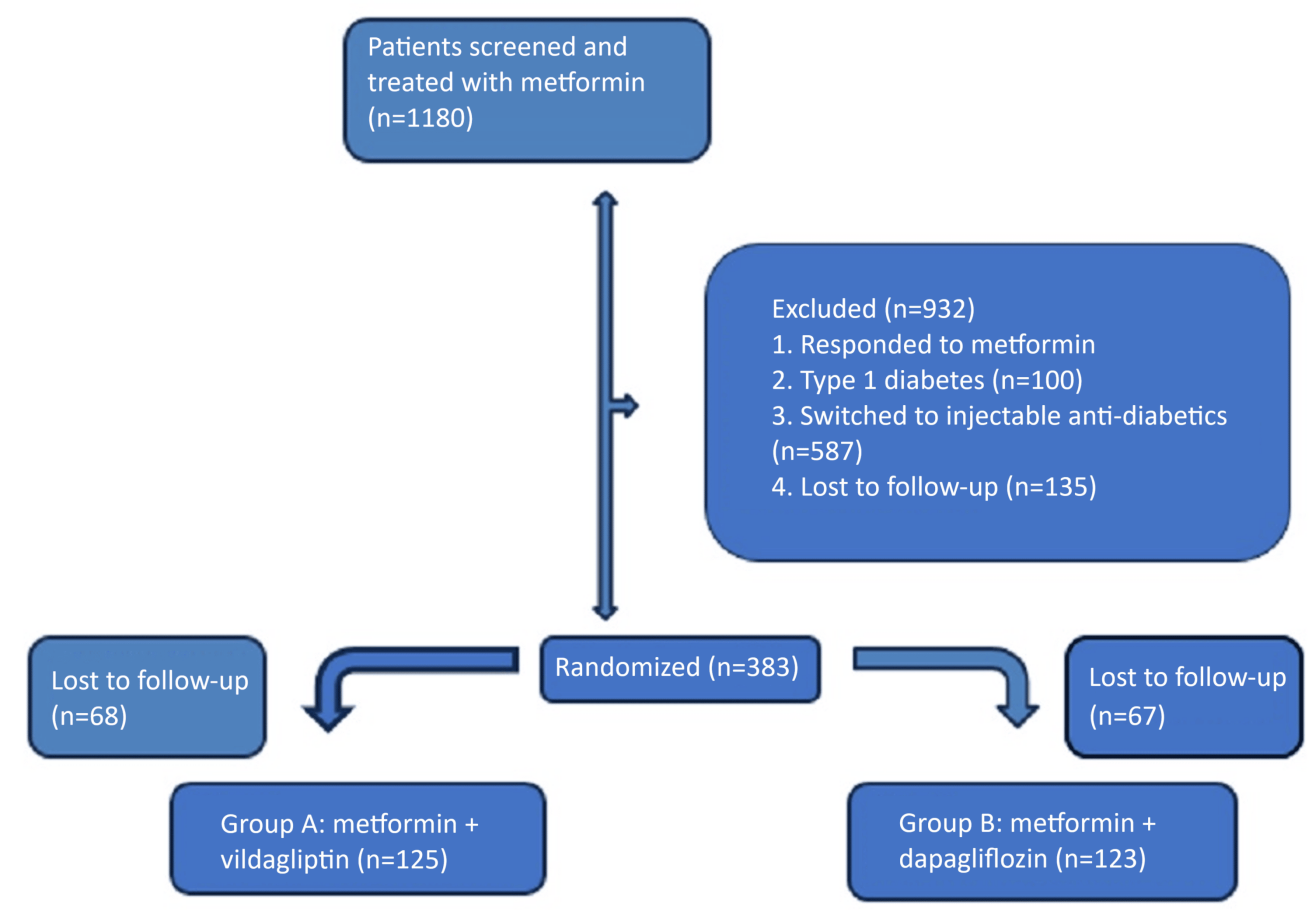
Flowchart of the study participants.

A total of 1180 patients were screened, out of which 383 patients were recruited. A total of 248 patients (21.01%) completed the study, and, according to the per-protocol (PP) analysis, were included in the final efficacy assessments (Figure 1). Among 248 patients, 125 were prescribed metformin and vildagliptin, and 123 were prescribed metformin and dapagliflozin. The mean age of participants was 53.8±8.4 years, and the mean BMI was 25.0±3.4 kg/m^2 ^(Table 1).

**Table 1:** Baseline sociodemographic and clinical parameters of the study population. CCB’s: calcium channel blockers; ACEI’s: angiotensin-converting enzyme inhibitors; ARBs: angiotensin receptor blockers; eGFR: estimated glomerular filtration rate; ALT: alanine transaminase; AST: aspartate aminotransferase; IQR: interquartile range

Variables	Total	Metformin+vildagliptin	Metformin+dapagliflozin	p-Value
Number	248	125	123	0.687
Women	108 (43.5%)	55	53	0.741
Age (mean±SD)	53.8±8.4	52.3±5.7	54.7±6.4	0.689
Duration of diabetes (years), median (IQR)	2.5 (1.04-4.23)	2.7 (1.85-4.10)	2.4 (1.70-2.81)	0.453
Hypertension (n)	84	41	43	0.985
Smoking (n)	45	22	23	0.089
BMI (kg/m^2^) (mean±SD)	25.0±3.4	24.6±6.1	27.8±7.8	0.632
Diabetic retinopathy, n (%)	4 (1.6%)	2 (0.8%)	2 (0.8%)	0.563
Diabetic neuropathy, n (%)	12 (4.8%)	5 (2.01%)	7 (2.8%)	0.845
Fasting plasma glucose (mean±SD)	172.1±14.2	168.2±26.5	181.8±9.4	0.753
Baseline HbA1c (mean±SD)	8.52±0.65	8.44±0.57	8.86±0.24	0.542
eGFR (mL/min/1.73 m^2^), median (IQR)	91.5 (82.7-94.4)	94.1 (90.4-97.4)	90.6 (88.9-95.2)	0.865
Baseline ALT (mean±SD)	45.1±7.2	43.6±5.3	47.4±6.9	0.887
Baseline AST (mean±SD)	43.5±4.5	42.7±5.2	44.9±6.1	0.956
Previous treatment metformin, n (%)	248 (100)	125 (100)	123 (100)	-
CCB’s/ACEI’s/ARBs, n (%)	74 (29.8%)	38 (15.3%)	36 (14.5%)	0.879

There were no statistically significant differences in age, sex, duration of diabetes, and other baseline laboratory findings, including the FPG, HbA1c levels, and the BMI between the two groups. During the follow-up, the mean FPG levels in Group A (metformin with vildagliptin) were reduced by 16.6 mg/dL (95% CI: -21.7 to -7.4; p<0.05). The mean HbA1c levels in Group A decreased by 0.71% (95% CI: -0.84 to -0.55) from 8.44±0.57% to 7.73±0.37% (p<0.05).

The mean change in the levels of TC after 24 weeks of treatment in group A was significantly reduced by 19.3 mg/dL (95% CI: -2.1, -25.8) (p=0.011), the plasma LDL-C decreased from 101.6±28.6 to 89.8±33.9 mg/dL (95% CI: 2.10, 16.6) (p=0.03), whereas, the decrease in the plasma TG levels by 6.4 mg/dL (p=0.37) and an increase in the HDL-C levels by 1.1 mg/dL were not statistically significant (p=0.78) (Table 2).

**Table 2:** Effects of metformin and vildagliptin on metabolic parameters. *P<0.05 (comparison between before treatment and after treatment in all subjects). **P<0.05 (comparison between before treatment and after treatment in each group). ***P<0.05 (comparison between DPP-4 inhibitor group and SGLT2 inhibitor group). Data are presented as means±SD or 95% CI. P<0.05 was considered significant. Changes from baseline and percentage change from baseline are adjusted for age, sex, diabetes duration, BMI, and glucose control status (HbA1c difference). DPP-4: dipeptidyl peptidase 4; SGLT2: sodium glucose co-transporter 2; LDL: low-density lipoprotein; HDL: high-density lipoprotein; HbA1c: hemoglobin A1c

Parameters	Total			Metformin+vildagliptin			p-Value
	Baseline	24 Weeks		Baseline		24 Weeks	
Fasting blood glucose (mg/dL)	172.1±14.2	150.7±15.6*		168.2±26.5		151.6±21.3**	0.59
Change (95% CI)	-21.34 (-11.42, -24.86)			-16.6 (-7.4, -21.7)			
HbA1c (%)	8.62±0.60	8.16±0.32		8.44±0.57		7.73±0.37	0.65
Change (95% CI)	-0.46 (-0.27, 0.52)			-0.71 (-0.55, -0.84)			
Total cholesterol (mg/dL)	192.6±21.1	176.1±12.6*		186.4±22.6		167.1±18.2**	0.87
Change (95% CI)	-16.5 (-12.3, -21.56)			-19.3 (-2.1, -25.8)			
LDL-cholesterol (mg/dL)	105.5±13.4	98.3±10.3*		101.6±28.6		89.8±33.9**	0.03
Change (95% CI)	-7.2 (-3.9, -6.4)			-11.8 (-2.10, -16.6)			
HDL-cholesterol (mg/dL)	40.1±4.5	42.5±3.2		40.1±4.3		41.2±4.5	0.78
Change (95% CI)	2.4 (2.1, 5.2)			1.1 (0.71, 2.12)			
Triglycerides (mg/dL)	179.7±29.5	173.3±25.4*		179.0±33.9		172.6±28.6	0.37
Change (95% CI)	-6.4 (4.2, 11.4)			-6.4 (4.10, 10.69)			
Systolic blood pressure (mmHg)	127.6±12.45	125.3±16.73		127.2±12.7		125.7±12.6	0.65
Change (95% CI)	-2.3 (1.6, 3,4)			-1.5 (-0.32, -0.62)			
Diastolic blood pressure (mmHg)	78.3±4.67	77.9±5.23		77.5±6.3		77.1±6.5	0.78
Change (95% CI)	-0.39 (0.85, 0.11)			-0.4 (-0.22, -0.54)			
Body weight (Kg)	76.12±3.45	74.98±12.56*		72.27±12.3		71.7±12.1	0.45***
Change (95% CI)	-1.14 (0.54, 1.45)			-0.57 (-0.15, -0.75)			
ALT	45.1±7.2		37.9±5.67	43.6±5.3		37.2±6.33	0.55
Change (95% CI)	-7.2 (3.56, 8.97)			-6.4 (3.89, 9.32)			
AST	43.5±4.5		37.5±4.65	42.7±5.2		36.0±5.45	0.39
Change (95% CI)	-6.0 (3.45, 8.97)			-6.7 (4.7, 8.9)			
Lipoprotein(a) (mg/dL)	18.9±9.8		17.9±7.9	16.8±8.7	16.4±9.3		0.767
Change (95% CI)	-1.0 (-0.54, -1.22)			-0.4 (-0.22, -0.61)			
Apolipoprotein A	134.6±23.8		137.8±32.5	135.5±34.2	139.8±37.9		0.986
Change (95% CI)	3.2 (1.8, 6.7)			4.3 (2.7, 6.5)			
Apolipoprotein B	82.3±23.8		78.5±24.3	89.8±27.5	86.7±23.8		0.563
Change (95% CI)	-3.8 (-1.4, 5.4)			-3.1 (-1.4, -5.3)			

The mean change in the levels of ALT after 24 weeks of treatment in group A decreased by 6.4 IU/L (p=0.55), and the mean change in the levels of AST decreased by 6.7 IU/L (p=0.39) (Table 2). After 24 weeks in group B (metformin and dapagliflozin), the mean FPG reduced by 23.5 (95% CI: -9.9, -31.1) mg/dL (p<0.05). The HbA1c levels reduced by -1.08% (95% CI: -0.86, -0.142) (p<0.05), i.e., lowered from 8.86±0.24% to 7.78±0.55% (Table 3).

**Table 3:** Effects of metformin and dapagliflozin on metabolic parameters. *P<0.05 (comparison between before treatment and after treatment in all subjects). **P<0.05 (comparison between before treatment and after treatment in each group). ***P<0.05 (comparison between DPP-4 inhibitor group and SGLT2 inhibitor group). Data are presented as means±SD or 95% CI. P<0.05 was considered significant. Changes from baseline and percent change from baseline are adjusted for age, sex, diabetes duration, BMI, and glucose control status (HbA1c difference). DPP-4: dipeptidyl peptidase 4; SGLT2: sodium glucose cotransporter 2; LDL: low-density lipoprotein; HDL: high-density lipoprotein; HbA1c: Hemoglobin A1c

Parameters	Total		Metformin+dapagliflozin		p-Value
	Baseline	24 Weeks	Baseline	24 Weeks	
Fasting blood glucose (mg/dL)	172.1±14.2	150.7±15.6*	181.8±9.4	157.9±10.2**	0.84
Change (95% CI)	-21.34 (-11.42, -24.86)		-23.5 (-9.9, -31.1)		
HbA1c (%)	8.62±0.60	8.16±0.32*	8.86±0.24	7.78±0.55**	0.67
Change (95% CI)	-0.46 (-0.27, -0.52)		-1.08 (-0.86, -1.42)		
Total cholesterol (mg/dL)	192.6±21.1	176.1±12.6*	194.3±23.9	185.6±17.8	0.58
Change (95% CI)	-16.5 (-12.3, -21.56)		-8.7 (-8.47, 9.32)		
LDL-cholesterol (mg/dL)	105.5±13.4	98.3±10.3*	99.4±14.6	105.8±16.7	0.04***
Change (95% CI)	-7.2 (-3.9, - 6.4)		+6.4 (6.10, 6.69)		
HDL-cholesterol (mg/dL)	40.1±4.5	42.5±3.2	40.2±4.8	43.37±5.1**	0.03***
Change (95% CI)	2.4 (2.1, 5.2)		+3.17 (2.01, 3.32)		
Triglycerides (mg/dL)	179.7±29.5	173.3±25.4*	179.82±32.1	173.4±24.4	0.87
Change (95% CI)	-6.4 (4.2, 11.4)		-6.4 (4.15, 7.69)		
Systolic blood pressure (mmHg)	127.6±12.45	125.3±16.73	134.1±12.7	132.8±11.1**	0.03
Change (95% CI)	-2.3 (-1.6, -3.4)		-1.3 (-0.8, -1.5)		
Diastolic blood pressure (mmHg)	78.34±4.67	77.95±5.23	79.2±6.3	77.8±5.9**	0.04
Change (95% CI)	-0.39 (-0.85, -0.11)		-1.4 (-1.0, -1.8)		
Body weight (kg)	76.12±3.45	74.98±2.56*	71.6±11.3	70.2±10.4**	<0.05***
Change (95% CI)	-1.14 (0.54, 1.45)		-1.4 (-0.8, -2.2)		
ALT	45.1±7.2	37.9±5.67	47.4±6.9	36.5±8.43	0.57
Change (95% CI)	-7.2 (3.56, 8.97)		-10.9 (5.5, 14.87)		
AST	43.5±4.5	37.5±4.65	44.9±6.1	39.4±7.94	0.63
Change (95% CI)	-6.0 (3.45, 8.97)		-5.5 (4.30, 7.65)		
Lipoprotein(a) (mg/dL)	18.9±9.8	17.9±7.9	19.2±8.9	17.7±8.52	0.76
Change (95% CI)	-1.0 (-0.54, -1.22)		-1.5 (0.94, 2.7)		
Apolipoprotein A	134.6±23.8	137.8±32.5	135.4±32.5	138.3±28.9	0.68
Change (95% CI)	3.4 (1.8, 6.7)		2.9 (1.8, 4.7)		
Apolipoprotein B	82.3±23.8	78.5±24.3	84.6±22.2	82.5±28.7	0.85
Change (95% CI)	-3.8 (-1.4, 5.4)		-2.1 (1.4, 3.4)		

The difference in the change in mean FPG (p=0.84) and mean HbA1c (p=0.67) levels between the DPP-4i and the SGLT2i was not statistically significant in this study. The plasma LDL-C levels in group B after 24 weeks increased by 6.4 mg/dL (95% CI: 6.10-6.69) (p=0.03). The mean plasma HDL-C levels increased by 3.17 mg/dL (95% CI: 2.01, 3.32) (p=0.03). The mean plasma TG in group B decreased by 6.4 mg/dL (95% CI: 4.15-7.69) (p=0.87). Whereas, the total cholesterol was reduced by 8.7 mg/dL (p=0.58) (Table 3). Statistically significant differences in the mean LDL-C and HDL-C levels after 24 weeks of therapy were observed between the two groups.

The change in the lipid profile between the vildagliptin group and the dapagliflozin group showed a significant difference in HDL-C (p=0.03) and LDL-C (p=0.04) after analysis using ANCOVA, with adjustments for age, gender, duration of diabetes, BMI, and change in HbA1c levels. The mean change in the levels of ALT after 24 weeks of treatment in group B decreased by 10.9 (p=0.57), and the mean change in the levels of AST after 24 weeks decreased by 5.5 mg/dL (p=0.63). Additionally, a statistically significant decrease in the blood pressure levels and body weight from baseline to 24 weeks was observed in group B (Table 3).

## Discussion

This observational prospective study establishes that vildagliptin or dapagliflozin, in combination with metformin, have varying effects on the metabolic profile in patients with uncontrolled T2D. Inadequately controlled T2D is an independent risk factor for dyslipidemia and its associated outcomes. Dyslipidemia hastens the onset and progression of atherosclerosis, thereby increasing the risk of ASCVD [12].

The mean changes in mean HbA1c levels from baseline in both the groups (p=0.76) were in line with the study by Del Prato et al. that highlighted the combination of an SGLT2i and metformin to have better efficacy in long-term control of plasma glucose levels and mean HbA1c compared to the combination of a DPP-4i and metformin [13]. These observations were in contrast with meta-analyses by Wang et al., where the combination of metformin and DPP-4i was more effective compared to the combination of metformin and SGLT2i on the reduction of mean FPG and the mean HbA1c levels [14]. The mean change in mean FPG levels from baseline in the vildagliptin and dapagliflozin groups (p=0.068) was consistent with the study by Kumar [15].

In this 24-week study, we observed that the addition of vildagliptin to metformin improved the mean plasma HbA1c levels, mean plasma TG, and significantly reduced the mean FPG and the mean TC levels. In another similar study, vildagliptin was reported to have matching beneficial effects on mean plasma TC, mean plasma TG, and plasma glucose in patients with T2D [16]. Whereas, after 24 weeks of therapy with the combination of metformin and dapagliflozin, the mean FPG levels, mean HbA1c levels, and the mean HDL-C and mean LDL-C levels were statistically significantly decreased compared to metformin and vildagliptin combination therapy. Similar findings have been reported in a study done by Bays et al. [17].

In the present study, although the change in the mean HDL-C levels in both groups after 24 weeks showed considerable improvement, the effect of metformin and dapagliflozin combination on HDL-C was statistically significant. Consistent findings have been reported in another systematic review and meta-analysis [18]. Additionally, there was a statistically significant reduction in blood pressure and body weight in the metformin and dapagliflozin group after 24 weeks of therapy. These findings are consistent with another study done by Zhang et al. [19].

This study advocates the use of dapagliflozin in T2D patients with low HDL-C levels. Additionally, the present study found that the mean LDL-C levels increased in both groups. However, the metformin and dapagliflozin combination group reported a significant increase in the LDL-C levels compared to the metformin and vildagliptin group. A similar significant increase in LDL-C levels has been reported in a meta-analysis by Martha et al. [20].

In the present study, although non-significant, there was a considerable decline in liver transaminase levels in group B. This suggests a possible shielding effect of dapagliflozin on fatty liver disease that tends to occur concomitantly with T2D. A recent similar study highlighted a beneficial association between SGLT2i use and improvements in transaminase levels and overall hepatic functioning [18].

Limitations

This study had a few limitations. First, the study was conducted in a single center. Second, the duration of follow-up was short. Third, the sample size was small, which could have introduced confounding effects on the study's results. Fourth, the change of medication during the study period was not permitted; therefore, we excluded patients who had changed medications during the follow-up period. Further long-term studies are needed to replicate the results of this study in the future.

## Conclusions

In conclusion, vildagliptin, a DPP-4i, and dapagliflozin, an SGLT2i, when added to metformin, have shown beneficial effects on glucose control and variable effects on lipid profiles in patients with inadequately controlled T2D. Although the addition of either vildagliptin or dapagliflozin to metformin was comparable, the addition of vildagliptin to metformin showcased a better reduction in the plasma LDL-C levels compared to the addition of dapagliflozin to metformin. Hence, the combination of vildagliptin and metformin may be better suited to patients with inadequately controlled T2D and dyslipidemia with raised plasma LDL-C levels. Thus, the addition of vildagliptin or dapagliflozin to metformin monotherapy in patients may be beneficial not only in regulating FPG and HbA1c levels but also lipid profile and overall reduction in the long-term associated risk of ASCVD in T2D patients with inadequately controlled plasma glucose levels.
